# Disruption of the moonlighting function of CTF18 in a patient with T-lymphopenia

**DOI:** 10.3389/fimmu.2025.1539848

**Published:** 2025-02-14

**Authors:** Robert Sertori, Billy Truong, Manoj K. Singh, Susan Shinton, Rachael Price, Andrew Sharo, Paulameena Shultes, Uma Sunderam, Sadhna Rana, Rajgopal Srinivasan, Sutapa Datta, Joan Font-Burgada, Steven E. Brenner, Jennifer M. Puck, David L. Wiest

**Affiliations:** ^1^ Nuclear Dynamics and Cancer Program, Fox Chase Cancer Center, Philadelphia, PA, United States; ^2^ Center for Computational Biology, University of California, Berkeley, Berkeley, CA, United States; ^3^ Innovation Labs, Tata Consultancy Services, Hyderabad, India; ^4^ Cancer Signaling and Microenvironment Program, Fox Chase Cancer Center, Philadelphia, PA, United States; ^5^ Department of Pediatrics, University of California, San Francisco, San Francisco, CA, United States; ^6^ Department of Pediatrics, University of California, San Francisco (UCSF) and UCSF Benioff Children’s Hospital, San Francisco, CA, United States

**Keywords:** T lymphocyte, thymus, immunodeficiency, zebrafish, CHTF18

## Abstract

**Introduction:**

Newborn screening for immunodeficiency has led to the identification of numerous cases for which the causal etiology is unknown.

**Methods:**

Here we report the diagnosis of T lymphopenia of unknown etiology in a male proband. Whole exome sequencing (WES) was employed to nominate candidate variants, which were then analyzed functionally in zebrafish and in mice bearing orthologous mutations.

**Results:**

WES revealed missense mutations in *CHTF18* that were inherited in an autosomal recessive manner. CTF18, encoded by the *CHTF18* gene, is a component of a secondary clamp loader, which is primarily thought to function by promoting DNA replication. We determined that the patient’s variants in *CHTF18* (CTF18 R751W and E851Q) were damaging to function and severely attenuated the capacity of CTF18 to support hematopoiesis and lymphoid development, strongly suggesting that they were responsible for his T lymphopenia; however, the function of CTF18 appeared to be unrelated to its role as a clamp loader. DNA-damage, expected when replication is impaired, was not evident by expression profiling in murine *Chtf18* mutant hematopoietic stem and progenitor cells (HSPC), nor was development of Ctf18-deficient progenitors rescued by p53 loss. Instead, we observed an expression signature suggesting disruption of HSPC positioning and migration. Indeed, the positioning of HSPC in *ctf18* morphant zebrafish embryos was perturbed, suggesting that HSPC function was impaired through disrupted positioning in hematopoietic organs.

**Discussion:**

Accordingly, we propose that T lymphopenia in our patient resulted from disturbed cell-cell contacts and migration of HSPC, caused by a non-canonical function of CHTF18 in regulating gene expression.

## Introduction

Primary immune deficiencies are rare, with severe combined immunodeficiency (SCID), where T and B lymphocytes are absent or not functional, occurring in approximately 1/58,000 live births in the United States (1). The recently increased frequency of diagnosis is likely a result of newborn screening for SCID and T lymphopenia that is now occurring in all 50 states in the US (2–4). Newborn screening has allowed cases to be diagnosed and treated before maternal immunity has waned and infections occur, which has greatly improved the efficacy of treatment and led to identification of many new genes implicated in the etiology of SCID (3–5). Newborn screening relies on identification of reduced T cell receptor excision circles (TREC) in peripheral blood, which is a surrogate marker for impaired T cell output from the thymus (6, 7). This screening method has also identified many cases of T lymphopenia for which the causal etiology was initially unknown (8–10). Elucidating the causal etiology in such cases not only opens potential new avenues for the diagnosis and treatment of immunodeficiency diseases, but also represents an opportunity to identify novel regulators of lymphocyte development.

Unbiased investigation of the causal etiology of SCID or T lymphopenia cases can lead to the identification of genes of unknown function or identification of new functions for known genes. Candidate variant identification is pursued by whole exome sequencing (WES) followed by functional screening in zebrafish. Zebrafish are an attractive model for functional screening of candidate alleles, as they are amenable to genetic analysis and their hematopoietic processes, including lymphoid development, are highly conserved (11). For example, we previously employed this strategy to reveal a new function for STN1, a component of the CST complex (**C**TC1-**S**TN1-**T**EN1), which plays multiple roles in telomere maintenance specifically and genome stability generally (12, 13). Interestingly, the developmental anomalies produced by the *STN1* patient variant did not appear to result from impairing telomere maintenance or genome integrity, as the defects were rescued by treatment with Thalidomide, and were not phenocopied by knockdown of another CST complex component, CTC1 (Unpublished data) (14). Finally, we have also identified mutations in genes critical for essential processes, such as actin remodeling (*ARPC1B)*, transcription (*MED14)* or clamp loader function *(CHTF18) (* 15 *;* 10 *)*, yet were compatible with live birth of immunodeficient patients.

Clamp loaders facilitate replication by loading onto DNA, a clamp comprising a trimer of proliferating cell nuclear antigen (PCNA), which is required for DNA replication (16, 17). PCNA is typically loaded onto DNA by the primary clamp loading complex, composed of five replication factor C (RFC) components, RFC1-5, which are essential for life in yeast and higher vertebrates (18, 19). In contrast, secondary clamp loader component, Chromosome Transmission Fidelity Factor 18 (CTF18), is not essential for life (20), either because it is a secondary clamp loader and/or because its expression is more tissue restricted than primary clamp loader component RFC1 (biogps/human atlas).

Here we describe our investigation of the causal etiology in an infant whose T lymphopenia was detected by newborn screening. Functional screening in zebrafish revealed that missense mutations in the secondary clamp loader component *CHTF18*, were potentially responsible for disease in the proband. Despite the prevailing view that the predominant function of CTF18 (encoded by the *CHTF18* gene) is facilitating DNA replication as part of a clamp loader, our data suggest its role in supporting hematopoiesis may be unrelated to this activity. Indeed, Ctf18 loss produced no evidence of DNA-damage, and did not impair proliferation; however, it did cause selective changes in the expression of genes in hematopoietic stem and progenitor cells (HSPC) that influence cell-cell interactions and migration, suggesting that *CHTF18* mutant HSPC would make suboptimal contacts with supporting stroma and fail to execute the prescribed migration patterns required or successful hematopoiesis. Thus, we propose that CTF18 supports normal hematopoiesis and lymphopoiesis through a “moonlighting function” unrelated to the support of DNA replication, but rather controls proper expression of genes regulating HSPC positioning and migration.

## Materials and methods

### Patient information

T lymphopenia was identified in the male proband by routine newborn SCID screening for T cell receptor excision circles (TREC) using blood from a Guthrie blood spot filter card, as described (1, 21, 22). Research activities were performed with parental informed consent under protocols approved by the institutional review board (IRB) at the University of California, San Francisco.

Research-based whole exome sequencing (WES) was performed on cells from the proband and parents with bioinformatic analysis and variant calling, as described (9, 23). Briefly, libraries were prepared by appending TruSeq adaptors (Illumina: San Diego, CA) to genomic DNA fragments of 200–300 bp that were enriched with 10 cycles of PCR, pooled and submitted to exon capture using a Roche Nimblegen kit (V3.0). After 10 additional amplification cycles, 100 bp paired end sequence reads were generated (HiSeq2000, llumina) and aligned against GRCh37 (Aug 2009 release) using BWA (v0.6.2). The results were converted to BAM, sorted by coordinate, indexed, and marked for PCR duplicate reads using the Picard toolkit (v 1.81) (http://picard.sourceforge.net). Local realignment was performed around known indel locations, and base quality scores were re-calibrated using GATK (v 2.6.5). Variants were called using GATK UnifiedGenotyper and freebayes (vesion 0.9.10) and variant quality scores were re-calibrated by VQSR (Variant Quality Score Recalibration) using the trio of exomes in this report, as well as 65 others sequenced at our site. HapMap v3.3, 1000 genomes high confidence SNPs (phase1 v3 2010-11-23) and Omni chip array sets were used as training data, and to provided truth sites for SNPs, while the Mills dataset from the 1000 genomes was used for indels, using a truth sensitivity cutoff of 99% (24). Variant annotation (including region, effect, allele frequency, disease phenotype annotation, and conservation) was performed using our custom tool Varant (http://compbio.berkeley.edu/proj/varant/). Particular attention was given to high-confidence, rare, likely-damaging, protein-altering variants in genes associated with primary cellular immunodeficiency (25). WES data from the JPSCID-15 trio were submitted to dbGaP, accession number phs002968.v1.p1.

### Zebrafish

Tubingen long fin zebrafish were maintained at 28.5°C under standard aquaculture conditions. Animal housing and handling were all performed in accordance with the approved protocol from the Institutional Animal Care and Use Committee (IACUC).

### Analysis of candidate variants in zebrafish

The zebrafish *CHTF18* ortholog *chtf18* (NM_001110102.3) was previously identified. *CTBP2* paralogues ctbp2a (NM_001195491.1 and NM_131715.1), and ctbp2l (NM_001015064.1) were identified by homology and synteny, as described (10, 26). Antisense morpholino oligonucleotides (MO; Gene Tools) were used to block splicing of zebrafish *chtf18* (5’ ACTGGAACAAAACACACCTCTTCAT 3’), ctbp2a (5’ TTCGGTCAGAAACAATCATACACAC 3’) and ctbp2l (5’ CCGGAAACAAGTAATGATGCAACTG 3’) upon injection into one-cell embryos using a PM1000 microinjector (Microdata Instrument, Inc.). The efficacy of the MO in disrupting splicing was assessed by reverse-transcriptase (RT)–PCR using the following oligonucleotides (**Table 1**): 1) *chtf18*: F-AGGAGGCAAGATGTGGCG and R-CATTTCAGCAGACAGCGATTAG; 2) *ctbp2a:* F-CGGTGGGTGCGATGATG and R-TGTACCAGGCTGTGTGAGGG; 3) *ctbp2l*: F-CAGGAGCGGCTCCGAAGACGTTTTCCGGC and R-ACAGAAGGCAACGGTTGCCAGATC; and 4) *actb*: F-TGGCATCACACCTTCTAC and R-AGACCATCACCAGAGTCC (27). Tp53 rescue experiments were conducted as described in (28). To assess the capacity of wildtype and variant human *CHTF18* orthologs to rescue the loss of zebrafish, wildtype and patient variant *CHTF18* were cloned into heat shock-inducible expression vector pSGH2 using standard approaches. Sanger sequencing confirmed the R751W, V776L and E851Q variant alleles. Ectopic expression of wild-type and mutant human *CHTF18* was achieved by injection of the heat-inducible pSGH2 vector (29) into one-cell stage embryos, which were heated to 37°C for 1 hour at 30 hours post fertilization. GFP+ embryos were selected at 5 dpf for analysis by WISH using a probe for *lck* as described (9). The impact of *chtf18* knockdown on HSPC localization was assessed by injection of *chtf18e6* splice blocking MO followed by the performance of WISH at 36 hours post fertilization, as described (28).

**Table 1:** Oligonucleotides.

Primer	Target	Sequence
R745W sgRNA	*Chtf18*	TGGTGTGGCCCGGCTACGCG
R745W HDR oligo	*Chtf18*	ACTCGGATGAGCCAGACAAGGAACCACATACAGACACT GGTGTCAGGTATGGCACCGACTACGCGTAGCTGGGCCA CACCACAGGCCCTTGTTCTAGATACTCTCTGCCTGCTCCTG GATGTCCTCGCACCCAAGCTGCGCCCCGTGAGt
E845W sgRNA	*Chtf18*	TATTGCTCGGGAGATTGAAA
E845W HDR oligo	*Chtf18*	CCTGAACTACCTGCCCGAAAGCCCCTCACCTACCAGGCTAAGCAGCTTATTGCTCGGGAGATTGAAATGCAGAAGATGCGCAGGGCAGAGGCTTTGGCCTGGGCTCGAAGTGGCCCCCAGGTaagtttgtccccggtgttgagAcagcat
R751W mutagenesis	*CHTF18*	CGCCAGCCACGCGCAGCTGGGCCACGCCCCAGGCCC
R751W mutagenesis	*CHTF18*	GGGCCTGGGGCGTGGCCCAGCTGCGCGTGGCTGGCG
V776L mutagenesis	*CHTF18*	CACCCAAGCTCCGCCCCTTGAGCACACAGCTGTA
V776L mutagenesis	*CHTF18*	TACAGCTGTGTGCTCAAGGGGCGGAGCTTGGGTG
E851Q mutagenesis	*CHTF18*	CCCGCGAGATCGAGGTGCAGAAGATGCGGCGGGCG
E851Q mutagenesis	*CHTF18*	CGCCCGCCGCATCTTCTGCACCTCGATCTCGCGGG
R745W genotyping-F	*Chtf18*	CTAGGCCCAGACTCGGATGA
R745W genotyping-R	*Chtf18*	TACCCACAAGGCAGGACAAC
E845W genotyping	*Chtf18*	GTATCTTGCCTCACCCACCTG
E845W genotyping	*Chtf18*	CAAACCACTGATGTTGTATGCTG
*chtf18e6 MO*	*chtf18*	ACTGGAACAAAACACACCTCTTCAT
*ctbp2a MO*	*ctbp2a*	TTCGGTCAGAAACAATCATACACAC
*ctbp2al MO*	*ctbp2a*	CCGGAAACAAGTAATGATGCAACTG
*chtf18* PCR forward	*chtf18*	AGGAGGCAAGATGTGGCG
*chtf18* PCR reverse	*chtf18*	CATTTCAGCAGACAGCGATTAG
*ctbp2a* PCR forward	*ctbp2a*	CGGTGGGTGCGATGATG
*ctbp2a* PCR reverse	*ctbp2a*	TGTACCAGGCTGTGTGAGGG
*ctbp2l* PCR forward	*ctbp2l*	CAGGAGCGGCTCCGAAGACGTTTTCCGGC
*ctbp2l* PCR reverse	*ctbp2l*	ACAGAAGGCAACGGTTGCCAGATC
*actb* PCR forward	*actb2*	TGGCATCACACCTTCTAC
*actb* PCR reverse	*actb2*	AGACCATCACCAGAGTCC

### WISH analysis

WISH was performed as described (30) using anti-sense probes for *lck, ikaros, tcrd, runx1*, and *foxn1* (28, 31). The stained embryos were photographed using the Nikon SMZ1500 stereomicroscope equipped with DS-Fi1 digital camera and Nikon Ar imaging software. Image J software was used to measure integrated density of the *lck* WISH staining of zebrafish thymi.

### Structural modeling

Genomic sequences were obtained using the NCBI and ENSEMBL databases. Multiple alignments of human, mouse and zebrafish CTF18 and CTBP2 amino acid sequences were obtained using Clustal X. Structural modeling was performed as described for human CTF18 using UCSF Chimera software and AlphaFold predicted structures for CTF18 wildtype, the arginine 751 to tryptophan (R751W) variant and glutamic acid 851 to glutamine (E851Q) variant, as well as the RFC1 clamp loading structure (PDB code 6VVO) (10, 32). CTF18 has limited homology to the clamp loader paralog RFC1 found in the CryoEM structure of human clamp loader bound to PCNA (32). AlphaFold-Multimer v2.3 in the ColabFold implementation (33) was used to predict the structure of a conserved portion of CTF18 (residues 595 to 865) that contained the 3 missense mutations identified in the proband and their interactions with the nearest neighbor subunit in the clamp loader complex, RFC3. The resulting heterodimeric models were subjected to a coordinate constrained method of protein relaxation using the Rosetta (34) molecular modeling suite to optimize side chain packing (35–37). These relaxed models were aligned by the RFC3 subunit using the UCSF Chimera 1.15 software package (38) and analyzed for contacts between CTF18 variants and RFC3 with its bound ADP. As a test to validate our modeling procedure, we used the same AlphaFold methods to recapitulate the RFC1-RFC3 interaction, and the resulting model superposed with RFC1 subunit of the 6VVO structure with an RMSD < 0.76 Å^2^ for 200 alpha carbons in this region. Final refined modeling methods utilized AlphaFold3 (AF3) (39), which can predict the combined structure of multicomponent complexes including proteins, nucleic acids, small molecules, ions and modified residues. We retained a focus on the conserved fragment of CTF18 (583-863) bound to RFC3, and added an ADP molecule and magnesium ion that is often bound in the active site. Multiple AF3 runs were used to generate 20 models each of the WT R751, mutant R751W, and the mutant E851Q. In all cases the models scored with high confidence (ipTM range 0.89 to 0.91) and backbone superposition of models yielded RMSD values in the range of 0.4 to 0.8 Å^2^. The WT R751 rotamer chosen by the AF3 modeling was the same as that found in the PDB structure 8UN0 (state7) for 17 of the top 20 models, albeit with very minor differences in position due to slight backbone variation. In all cases the top ranked models were used to analyze the interface interactions and prepare figures with UCSF Chimerax version 1.7.1. To obtain a general overall interface assessment, we also analyzed the CTF18(583-863)-RFC3 interface with PISA analysis (40).

### Mice

All mouse strains were housed in the Laboratory Animal Facility at Fox Chase, which is accredited by the Association for Assessment and Accreditation of Laboratory Animal Care, and handled in accordance with an IACUC approved protocol. Ctf18 R745W and E845Q knockin mutant mice were generated by CRISPR genome editing (10). Chf18 R745W mice were created using a 150 bp oligo donor to mutate R745 to W and silent cytosine (C) to thymine (T) to inactivate the PAM and prevent re-cutting of the repaired allele. Similarly, the Ctf18 E845Q mice were created using a 150 bp oligo to mutate E845 to Q and inactivate the PAM to prevent re-cutting of the repaired allele. The knockin mutation created BseYI and HpyCH4V restriction enzyme sites, respectively, to genotype the mutated alleles. All oligonucleotides used in study are listed in **Table 1**.

### Flow cytometry

Single-cell suspensions prepared from mouse thymus, spleen, bone marrow, or peripheral blood were stained with optimal amounts of the following fluorochrome-conjugated antibodies: anti-CD4 (GK1.5), anti-CD8 (53-6.7), anti-CD24 (M1/69), anti-CD25 (PC61), anti-CD44 (IM7), anti-CD62L (MEL-14), anti-CD69 (H1.2F3), anti-CD73 (TY/11.8), anti-CD90.2 (30-H12) anti-B220 (RA3-6B2), anti-NK1.1 (PK136), anti-TCRγδ (GL3), anti-TCRβ (H57-597), anti-CD19 (eBio1D3), anti-CD3 (17A2), anti-Gr1 (RB6-8C5), anti-Sca1 (D7), and anti-cKit (2B8). The antibodies were purchased from BD Biosciences, eBioscience, BioLegend, or Tonbo Biosciences. Dead cells were excluded from analyses using propidium iodide (PI). Data were analyzed on either a BD LSRII or Symphony A5 flow cytometer (BD Pharmingen) using Flowjo 9.96 software (Treestar, Inc).

### Single cell RNA-seq

30,000 lineage depleted bone marrow cells from wildtype or mutant mice were loaded with the objective of collecting a single library of 10,000 cells by Chromium controller (V3 Chemistry version, 10X Genomics Inc, San Francisco, USA). Barcoding and DNA purification were performed according to the manufacturer’s protocol. Sequencing was performed using 2 x 150 pair-end configuration on an NovaSeqX Plus platform at a sequencing depth of 375G/lane. Cell-barcode and unique molecular identifiers were extracted and aligned through Cell Ranger and expression analysis was performed using Seurat V4, as described (41). Uniform Manifold Approximation and Projection (UMAP) plots were used to visualize the data based on canonical component analysis of the top 1000 genes with highest dispersion between all sample groups. Gene ontology and Gene Set Enrichment analysis was performed using EnrichR.

### Transplant assays

CD45.1 mice were lethally irradiated with a total dose of 11 Gy, split in two doses of 6.5Gy and 4.5Gy, separated by three hours. After 24 hours, mice were injected *i.v.* in the tail vein with either 100,000 lineage depleted adult bone marrow (BM) CD45.2 hematopoietic progenitors alone or together with 100,000 adult BM CD45.1+ competitor progenitors delivered in 200μl Hank’s Balanced Salt Solution (HBSS), as described (10). Engraftment was monitored by retro-orbital bleeding at the indicated intervals. Mice were sacrificed after 6 weeks of engraftment and bone marrow, thymus and spleen were analyzed by flow cytometry. For fetal liver (FL) transplants, 100,000 embryonic day 14.5 (E14.5) FL cells from CD45.2 mice were transferred as above with 100,000 CD45.1 FL competitor cells as described above. Seeding assays were performed by transplanting adult BM progenitors as above and determining the seeding of the BM of recipient mice 4 days later by performing flow cytometry on explanted bone marrow cells for the allelic marker (CD45.2/1).

### HSPC function *in situ*

To assess the capacity of WT and compound *Chtf18* mutant HSPC to respond to repeated entry into cell cycle, mice were treated twice weekly for 60 days with two intraperitoneal doses of pIpC (15mg/kg) as described (42), with weekly monitoring of peripheral blood lymphocyte content by flow cytometry.

### *In vitro* cultures

E14.5 FL progenitors from WT and compound *Chtf18* mutant mice were cultured on OP9-DL1 monolayers, as described (43). Developmental progression was monitored by quantitating cell growth of control triplicate cultures or those treated with agents including Interferon-β, Mitomycin-C, Ganetespib, and Aramycin-C.

### Graphing and statistics

Graphic representations of data were generated using GraphPad prism V9, following statistical analysis using ANOVA and Student’s t tests. Significance values are indicated.

## Results

### Clinical history of a proband with T lymphopenia

The male infant (JPSCID-15) was born at term after an uncomplicated pregnancy to nonconsanguineous, healthy parents with no known family history of immune deficiency. Newborn screening revealed low T cell receptor excision circles (TREC; normal >25), with T-cell lymphopenia subsequently documented by flow cytometry (**Table 2**) (21). The proportion of naïve recent thymic emigrant (i.e., CD45RA+) CD4+ and CD8+ T cells was reduced, with CD8+ cells exhibiting a slightly greater reduction (available data shown in **Table 2**) (46, 47). B and NK cell numbers were normal. The patient had a normal physical exam with no syndromic features. Proliferation to phytohemagglutinin, immunoglobulin levels and titers to killed vaccines were obtained over the ensuing months and were normal (44, 45). During his first year the patient experienced one episode of pneumonia treated successfully with oral antibiotics, but no other infections. Growth and development remained normal until he was lost to follow-up after his second birthday. Genetic workup included normal fluorescent *in situ* hybridization for chromosome 22q11.2 deletion and normal alpha-fetoprotein that ruled out DiGeorge syndrome and ataxia telangiectasia, respectively. Sanger sequencing of a panel of SCID genes, including *ADA*, *DCLRE1C*, *IL2RG*, *IL7R*, *JAK3*, *RAG1*, *RAG2*, *RMRP*, and *ZAP70*, revealed no mutations.

**Table 2:** Patient Laboratory Test Values.

Analyte	Normal ranges*	Patient value, age 30 days	Patient value, age 1-2 years
WBC x 10^-3^/μl	9 (4-12)	5.3	4.4
Absolute lymphocyte count x 10^-3^/μl	4750 (2300-7600)	**2.0**	**2.3**
TREC/*ACTINB* copies per μl blood in dried blood spot	>18/>55	**7**/22,000	
CD3+ T cells/μl	2620 (1540-5063)	**669**	**547**
CD4+ T cells/μl	1923 (943-3650)	**562**	
CD8+ T cells/μl	667 (336-1482)	**86**	
CD19+ B cells/μl	893 (224-2594)	939	
CD16+/CD56+ NK cells/μl	334 (138-1204)	226	
% CD4+ T cells that were naïve (CD3+/CD4+/CD45RA+)	84% (67% to 93%)	74%	
% CD8+ T cells that were naïve (CD3+/CD8+/CD45RA+)	92% (78% to 100%)	100%	
IgG (mg/dl)	327-1270		657
IgA (mg/dl)	20-100		54
IgM (mg/dl)	21-215		116
Anti-pneumococcal antibodies, μg/ml, 13 serotypes	>1.3 protective^3^		13/13 protective
H. Influenzae antibody, μg/ml	>1.00		14.9
Tetanus toxoid antibody, IU/ml	>0.10		1.13

*Normal ranges (44, 45)

Bold values represent those outside of the normal range.

### Identification and vetting of patient variants found upon exome sequencing

While sequencing of known genes implicated in immunodeficiency revealed no mutations, whole exome sequencing (WES) revealed several potential variants, with the most likely variants being mutations in *CTBP2* and *CHTF18*. CTBP2 is a transcriptional corepressor which binds Proline-Isoleucine-Aspartate-Leucine-Serine (PIDLS) to repress target genes, including IL-2 (48, 49). In zebrafish, *ctbp2a/ribeye a* and *ctbp2l/ribeye b* are the major proteins in synaptic ribbons and loss of both genes leads to decreased electron density, synaptic localization and recruitment of calcium channels (50). The patient had two variants in *CTFBP2:* 1) *a de novo* CTFBP2 D437N missense mutation affecting an aspartic acid (D) residue conserved in mice and zebrafish Ctbp2a isoform 2, but not in Ctbp2a isoform 1 or Ctbp2l; and 2) a 12aa insertion following P389 in a portion of the protein conserved in all isoforms (**Supplementary Figure 1A**). To investigate the role of CTBP2 in T cell development, we employed morpholino oligonucleotides (MO) to knock down *ctbp2* expression in zebrafish embryos and assessed the impact on T cell development by performing whole mouse *in situ* hybridization (WISH) using a T cell specific *lck* probe at five days post fertilization (dpf; **Supplementary Figure 1B**). Splice site MO targeting *ctbp2a*, *ctbp2l*, alone or in combination attenuated *ctbp2a* and *ctbp2l* expression, but did not arrest in T cell development, suggesting that the *CTBP2* patient variants were not responsible for disease (**Supplementary Figure 1B**).

**Figure 1:**
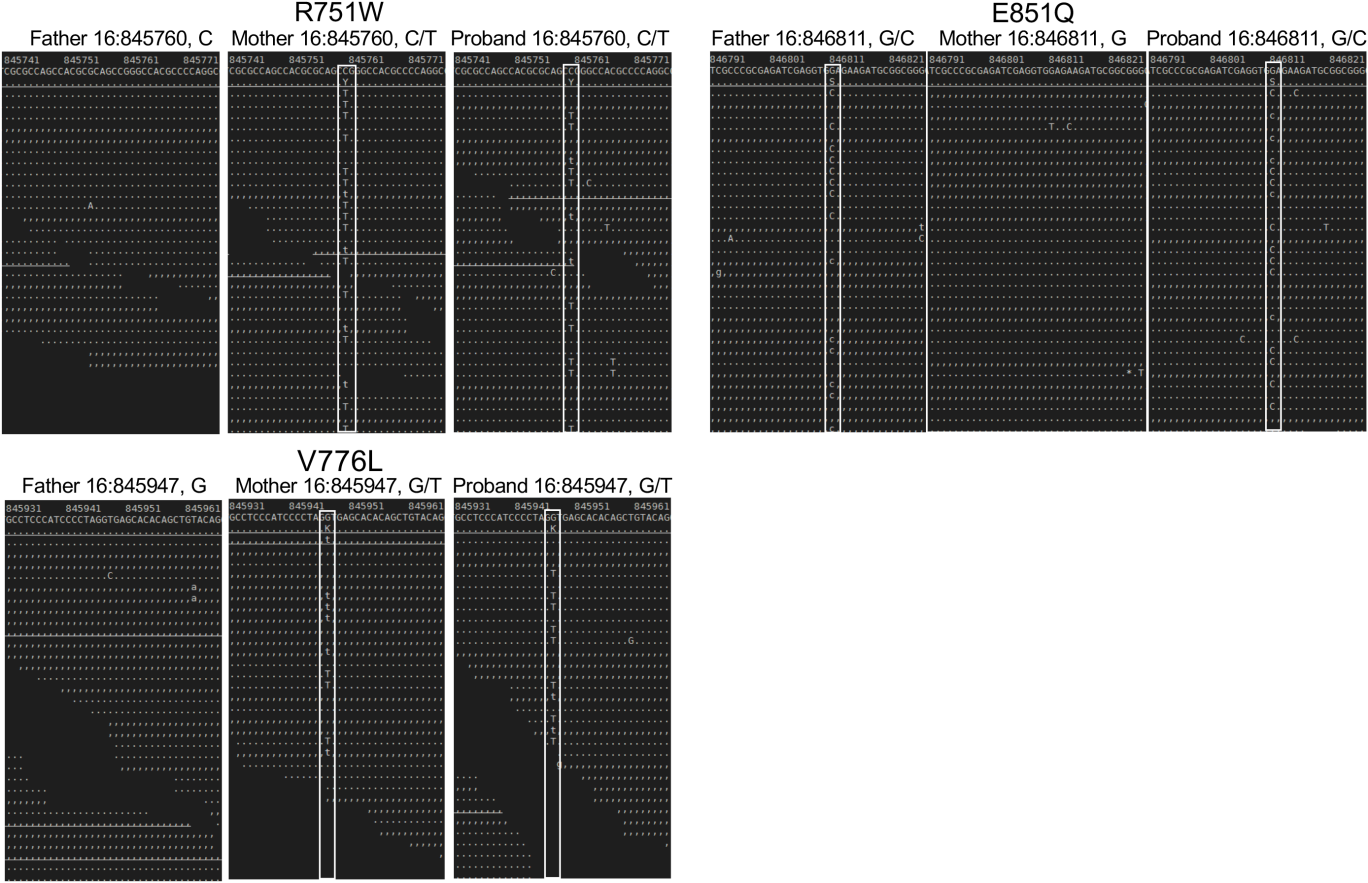
Sequence analysis of the parent and proband *CHTF18* alleles. NextGen sequence traces of the paternal, maternal, and proband *CHTF18* alleles are depicted, with the sequence variants boxed in white.

**Figure 2:**
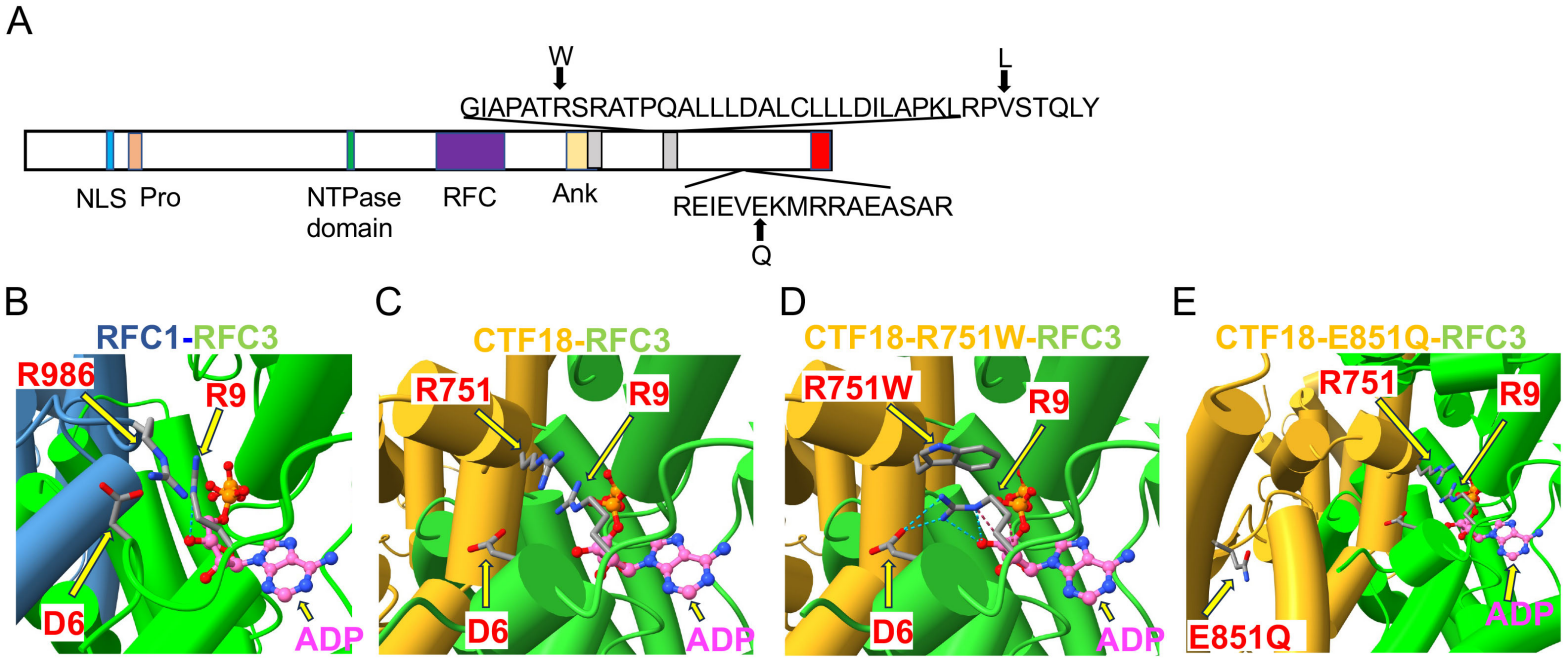
*Structural analysis of CHTF18 patient variants.* **(A)** The structure of human CTF18 is schematized with the structural domains and the location of variants inherited from the mother and father indicated above and below the diagram, respectively (52). NLS, nuclear localization signal; Pro, proline-rich domain; Ank, ankyrin repeat domain; TBD, triple barrel domain in red where CTF18 interacts with DSCC1 and CTF8 (53). **(B-E)** Clamp loader interface of either RFC1 or CTF18 with RFC3. **(B)** RFC1 structure (PDB #6VVO) shown in blue cylinders for helices and RFC3 in green. The ADP molecule (displayed with pink carbon atoms) bound by RFC3 is shown in ball-and-stick representation, and the key residues RFC1-986 and RFC3-R9 are labeled. **(C)** AF3 model of CTF18 (gold; residues 583-863) bound to RFC3. The wildtype R751 makes contact with RFC3-R9 in a similar manner compared to RFC1-R986. **(D)** AF3 model of the R751W variant makes more extensive contacts with RFC3-R9, changing the RFC-R9 rotamer conformation to where it makes 4 hydrogen bonds with RFC-D6 and the 3’ hydroxyl of ADP. **(E)** AF3 model of the CTF18-E851Q variant in a solvent exposed location distal from the RFC3 interface.

The only other highly ranked set of variants impacted the *CHTF18* gene. The CTF18 protein, encoded by the *CHTF18* gene, is a component of a secondary clamp loader complex, which loads PCNA onto DNA during replication (16, 17). CTF18 forms a pentamer with RFC2, RFC3, RFC4 and RFC5 (51). The proband exhibited two maternal (R751W and V776L) and one paternal (E851Q) missense variant in the human *CHTF18* gene, in residues that are conserved from human to mouse and zebrafish (**Figures 1**, **2A**; **Supplementary Figure 2**). To assess the extent to which these variants might disrupt the structure of the CTF18-containing secondary clamp loader, we examined a recently solved cryo-EM structure of the complex (51) and compared it to that of the RFC1-containing primary clamp loader complex using AlphaFold 2 (AF2) and AlphaFold 3 (AF3), with a particular focus on the impact of the CTF18 variants (**Figures 2B, C**) (32, 33, 54) (39). In the RFC1 complex, the RFC3-R9 residue is a key component of the ADP binding pocket and the ε amino group hydrogen bonds to the 3´ hydroxyl of ADP (**Figure 2B**). The analogous CTF18 loop, which is in close contact with RFC-R9, revealed a slightly different conformation containing a short alpha helix, as there are two extra amino acids in this CTF18 loop compared to the RFC1 loop (**Figure 2C**). Nevertheless, the analogous CTF18 residue to RFC1-R986, CTF18-R751, also points toward RFC3-R9 and the ADP binding pocket (**Figures 2B, C**; arrow), suggesting that it may also contribute to the dynamics of ADP binding and the ATP hydrolysis cycle. Moreover, the CryoEM structure led to speculation that the alternative CTF18 clamp loader complex is a much more mobile multi-subunit assembly compared to the canonical RFC1-based clamp loader. This may lower loading activity, thus limiting the alternative clamp loader complex to leading strand loading (51). Interestingly, the AF3 models of R751W variant forces a downward rotamer change for RFC3-R9 resulting in RFC3-R9 stably making four hydrogen bonds with either RFC3-D6 or the 3’hydroxyl of ADP (see blue dashed lines in **Figure 2D**, arrows indicate R751W and RFC3 R9). The R751W variant could cause a perturbation of the ATP hydrolysis cycle (i.e. slower ADP release) by rigidly fixing the rotamer of RFC3-R9. In contrast, the CTF18-E851Q variant is located away from the interface with RFC3 in a solvent exposed location and not near any other subunit in the CryoEM structures (**Figure 2E**, arrow indicates E851Q). This is a mild side chain substitution and it is unclear how this variant could alter function.

### Functional tests of CTF18 variants in zebrafish

**Figure 3:**
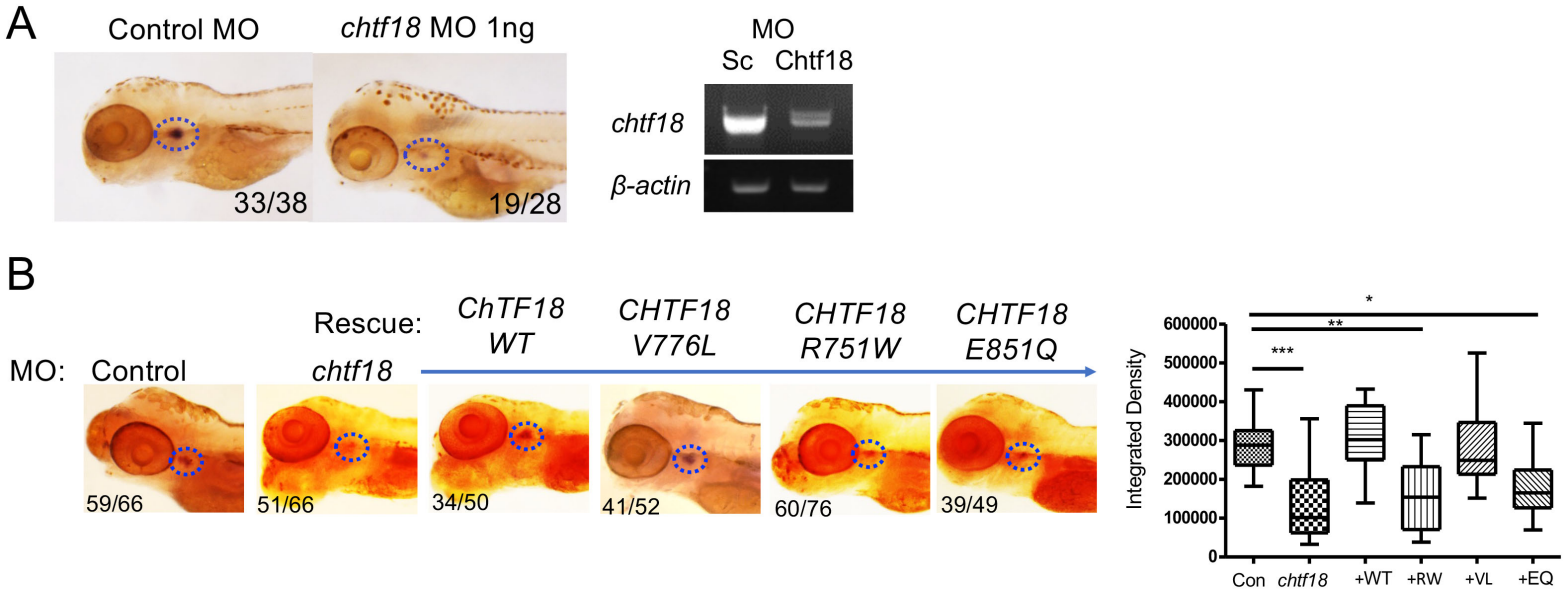
*CHTF18 patient variants fail to support T cell development.* **(A)** Effect of *chtf18* knockdown on T cell development at 5 days post fertilization (dpf) in zebrafish embryos. T cell development was assessed by whole-mount *in situ* hybridization (WISH) using a probe for the T cell specific kinase, *lck*. Numbers on images represent the fractions of embryos with the depicted phenotype. Thymus is indicated by dashed blue ovals. (right panel) The efficacy of *chtf18* E2I2 splice-blocking MO (1ng) was assessed by RT-PCR at 1 dpf. **(B)** Capacity of patient *CHTF18* variants to rescue T cell development in zebrafish. Lateral images of zebrafish treated with control or *chtf18* MO and subjected to rescue by heat-induced re-expression of the WT or mutant human *CHTF18* orthologs. Rescue of T-cell development was evaluated by *lck* WISH at 5 dpf. Thymus staining is indicated by dashed blue ovals and the fraction of embryos with the depicted phenotype is indicated on each micrograph. The intensity of the *lck* WISH signal was quantified by ImageJ software and is depicted graphically as box and whisker plots. Statistical significance was evaluated using one way ANOVA. *p < 0.05; **p < 0.01; ***p < 0.001.

To investigate the role of CTF18 protein in supporting T-cell development *in vivo*, we employed zebrafish, a tractable genetic model in which hematopoietic development is highly conserved (26, 55). Expression of the zebrafish *chtf18* ortholog was knocked down using a MO that interferes with *chtf18* sp*licing* and induces nonsense mediated decay (**Figure 3A**). The *chtf18* MO attenuated *chtf18* expression and markedly impaired T cell development at 5dpf, as indicated by decreased whole mount *in situ* hybridization (WISH) staining with an RNA probe for the T cell specific kinase, *lck* (**Figure 3A**). The blockade in T cell development caused by knockdown of *chtf18* indicated that Ctf18 performs an important function in supporting T cell development; however, it did not provide insight into whether the CTF18 patient variants damage CTF18 function. To determine whether the patient variants (R751W, V776L and E851Q) abrogated the function of CTF18 protein, we performed rescue experiments in zebrafish embryos. Endogenous *chtf18* was knocked down by MO as in **Figure 3A** and heat shock induction was employed to re-express the wild type and variant human *CHTF18* orthologs to determine if they rescue the block in T cell development caused by loss of zebrafish Ctf18 (**Figure 3B**) (9, 10). Re-expression of the wild type (WT) human ortholog compensated for the loss of Ctf18 and rescued T cell development, indicating that CTF18 function was conserved from human to zebrafish (**Figure 3B**). Moreover, the maternally inherited V776L variant also rescued development, indicating that this mutation is not damaging (**Figure 3B**); however, re-expression of either the R751W (maternal) or the E851Q (paternal) variant failed to rescue T cell development, indicating that each of these variants damage CTF18 function (**Figure 3B**). Taken together, these data suggest that T cell insufficiency in patient JPSCID-15 may result from compound heterozygosity of two damaging *CHTF18* mutations (R751W and E851Q).

### Loss of Chtf18 selectively blocks αβ T cell development in the thymus

To gain insight into the breadth and stage of developmental arrest caused by Ctf18 loss, we performed WISH using probes for other T cell subsets and thymic cell types. *Chtf18* knockdown did not appear to impair thymic seeding as staining for *ikaros*, a marker of early thymic progenitors, was not altered (**Supplementary Figure 3A**). The arrest of T cell development appeared to be restricted to αβ lineage T cells, since the γδ T cell subset identified using WISH with a *tcrd* probe was not affected by Ctf18 loss (**Supplementary Figure 3A**). Moreover, the block in αβ T cell development was likely to be cell autonomous, since WISH using a *foxn1* probe suggests that thymic stroma was not disrupted by *chtf18* knockdown (**Supplementary Figure 3A**). Finally, because CTF18 functions as a secondary clamp loader during DNA replication (17, 53, 56), we reasoned that *chtf18* knockdown might cause DNA damage resulting in a p53-dependent developmental arrest; however, *trp53* knockdown failed to rescue development in *chtf18* morphants, despite rescuing the p53-dependent arrest caused by knockdown of *rpl22* (**Supplementary Figure 3B**) (28). Together, these findings suggest that ctf18 loss arrests the development of αβ lineage T cells in a cell-autonomous, but p53-independent, manner.

### Generation of *Chtf18* mutant knockin mice

**Figure 4:**
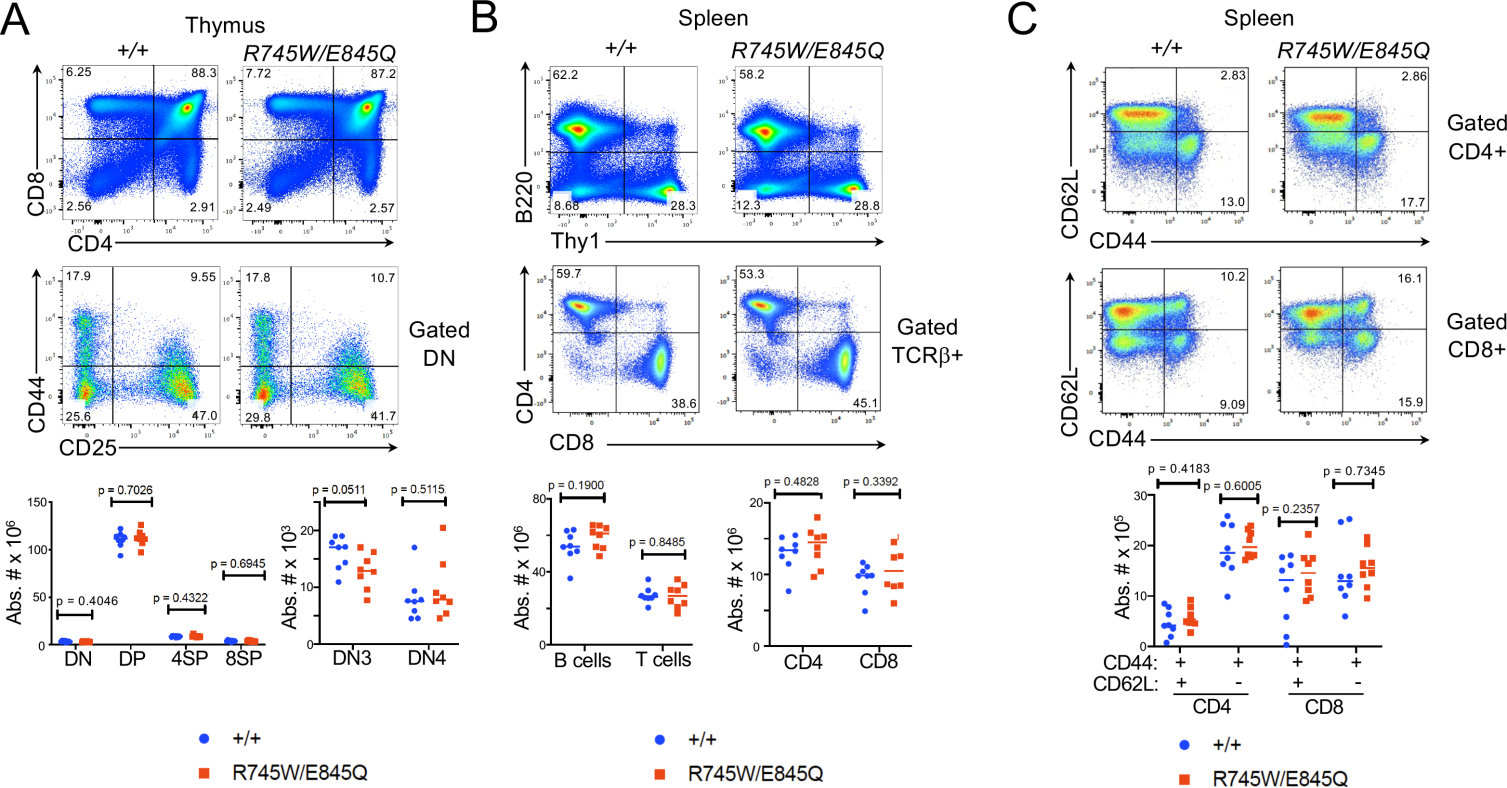
*Phenotype of Chtf18 mutant mice.* **(A-C)** Flow cytometric analysis of lymphoid development assessed by flow cytometry on thymus **(A)** and spleen **(B, C)**. Flow cytometry analysis was performed on explanted thymus and spleen from adult WT (+/+) and *Cht18* mut (*R745W/E845Q)* mice using the indicated antibodies to define the following populations in: **(A)** Thymus: CD4-CD8- (DN), CD4+CD8+ (DP), CD4+ (4SP), CD8 (8SP); **(B)** Spleen: B220+ (B cells); Thy1+ (T cells), CD4+, CD8+; **(C)** CD44+CD62L+ (effector memory); CD44+CD62L- (central memory). Absolute numbers of the indicated populations were depicted graphically as scatter plots with each symbol representing an individual animal. Statistical significance as assessed by multiple unpaired t tests.

**Figure 5:**
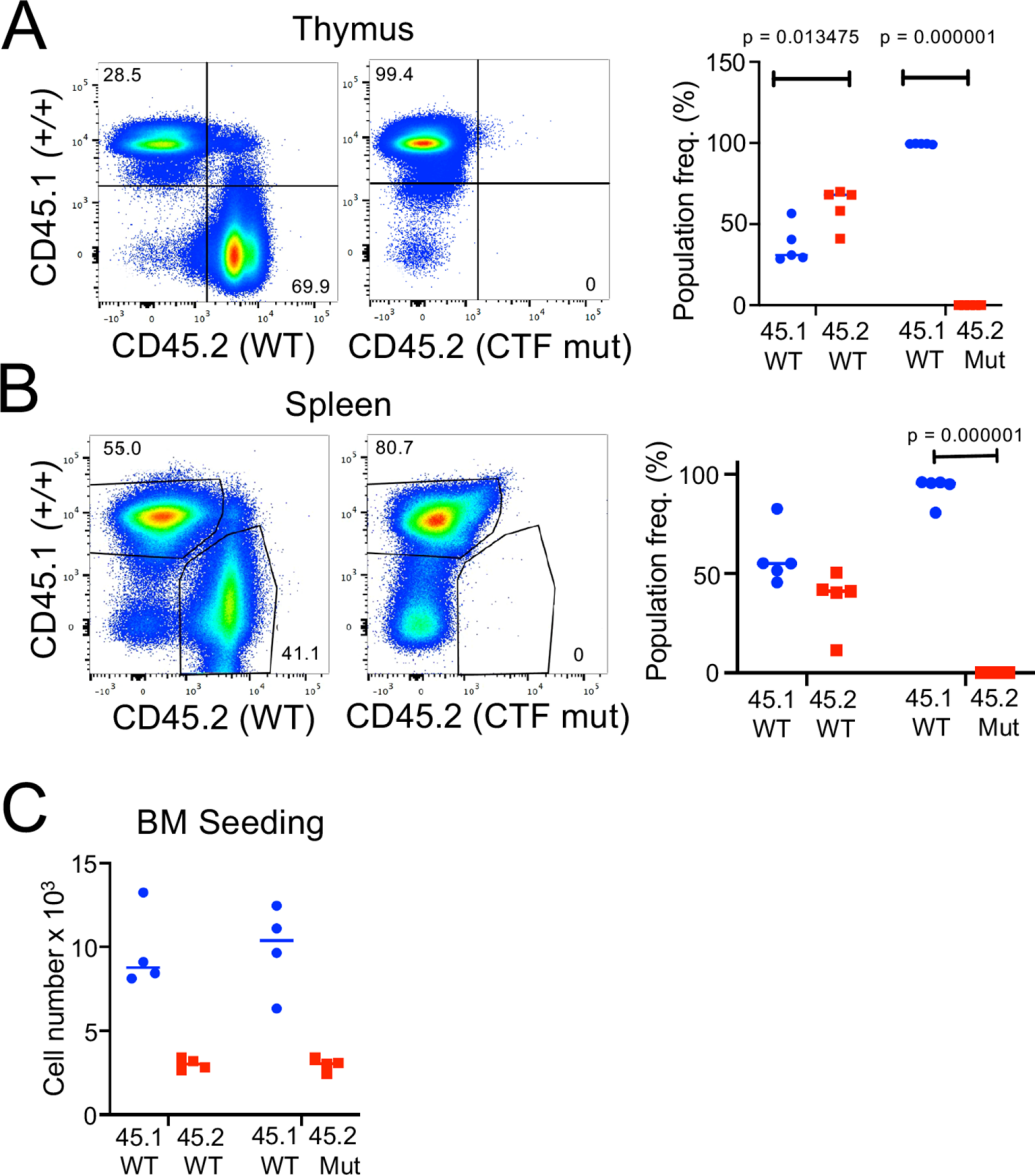
*Assessment of Chtf18 mut HSPC by competitive transplantation.* **(A, B)** The ability of adult *Chtf18* mut bone marrow HSPC to repopulate hematopoiesis was assessed by competitive transplantation using allotype marked (CD45.1) competitor. 100,000 Lineage^–^ HSPC from CD45.2 WT or *Chtf18* mut mice were mixed with an equal quantity of CD45.1 competitor cells and transferred into irradiated recipients. After 6 weeks, the hematopoietic reconstitution was assessed on explanted thymus **(A)** and spleen **(B)** cell suspensions by flow cytometry using anti-CD45.1 and anti-CD45.2 antibodies. Cell frequencies were displayed graphically as scatter plots. Statistical significance was assessed by multiple unpaired t tests. **(C)** The capacity of CD45.2 WT and *Chtf18* mut HSPC to home to the bone marrow was assessed as above expect that bone marrow seeding was measured by flow cytometry 4 days after transfer. The number of transferred cells found in the recipient bone marrow was exhibited by scatter plot. Statistical significance was assessed by multiple unpaired t tests.

To understand the mechanistic basis for Ctf18 action, we generated knockin mice bearing patient variants in the orthologous residues of mouse Ctf18, R745W and E845Q (**Supplementary Figure 4**). The knockin mice were generated using CRISPR-induced cutting and HDR oligos for repair. The resulting compound heterozygous *Chtf18^R745W/E845Q^* (*Chtf18* mut*)* mice were fertile and viable, with no outward signs of abnormalities (data not shown). We next examined T cell development by flow cytometry (**Figure 4A**). We observed no significant difference in any of the thymic subsets defined by CD4, CD8, CD44, and CD25. Moreover, splenic T cell numbers were not diminished; nor did they exhibit any signs of homeostatic proliferation marked by changes in expression of CD62L and CD44 (**Figures 4B, C**) (57). Developmental defects in some cases are evident only when hematopoietic stem and progenitor cells (HSPC) are stressed (10, 58). To place *Chtf18* mut HSPC under stress, we performed mixed bone marrow chimera analysis using allotype marked (CD45.1) competitor cells (**Figure 5A**). While the wild type *Chtf18+/+* (WT) HSPC effectively reconstituted the thymus and spleen of recipient mice, the *Chtf18* mut HSPC were unable to reconstitute recipient mice (**Figures 5A, B**). The failure to reconstitute recipient mice did not result from failure to home to the bone marrow, as a seeding assay revealed no defect in *Chtf18* mut HSPC reaching the bone marrow of the recipients (**Figure 5C**). Together, these data indicate that *Chtf18* mut HSPC are able to support hematopoiesis at baseline, but fail to do so under stress during competitive bone marrow transplantation.

### Transcriptomic analysis of *Chtf18* mutant HSPC

**Figure 6:**
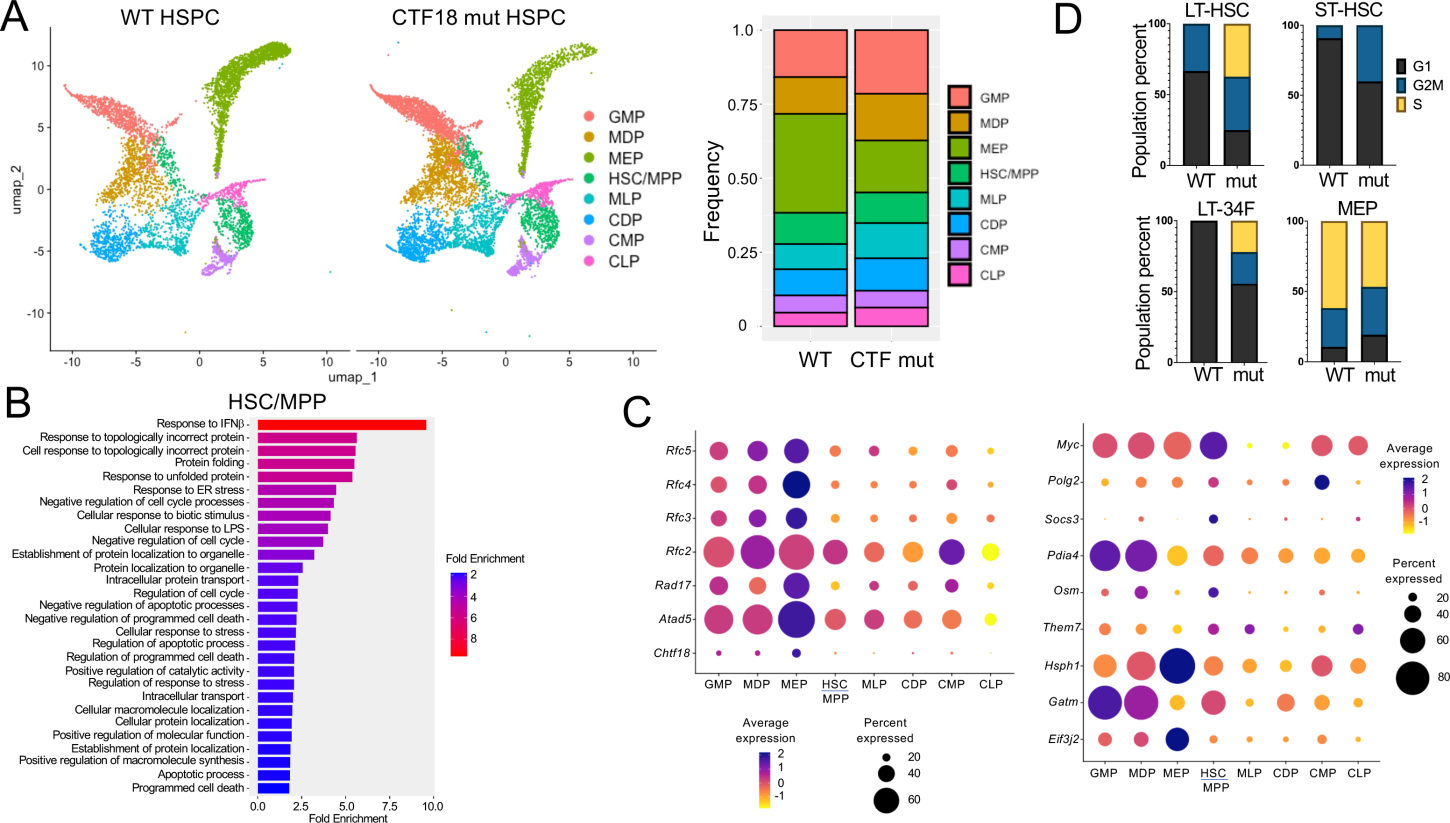
*scRNA-Seq analysis of WT and Chtf18 mut HSPC.* **(A)** Uniform Manifold Approximation Projection (UMAP) plots showing eight distinct HSPC clusters and their relative frequencies in Lineage- HSPC from *Chtf18* wildtype and mut mice. **(B)** Gene Set Enrichment analysis (EnrichGo) of the highly variable expressed genes between *Chtf18* wildtype and mut HSC/MPP cluster. **(C)** Bubble plot representation showing expression of DNA clamp loader proteins and top upregulated genes in the *Chtf18* mut HSPCs. **(D)** Percentage of cells in G1, G2/M and S phase in *Chtf18* WT and mut HSPCs. Reference cell type abbreviations: LT-34F, CD34+ Long-term reconstituting stem cell; LT-HSC, CD34- Long-term reconstituting stem cell; MEP, Megakaryocyte-Erythroid Progenitor; CMP, Common Myeloid Progenitor; GMP, Granulocyte-Monocyte Progenitor; MLP, Multilineage Progenitor; HSC/MPP, Hematopoietic Stem Cell and Multi-Potent Progenitor; CLP, Common Lymphoid Progenitor; MDP, Monocyte-Dendritic cell Progenitor; CDP, Common Dendritic cell Progenitor.

To elucidate the molecular basis for the impaired capacity of *Chtf18* mut HSPC to reconstitute hematopoiesis in competitive transplant experiments, we performed single-cell RNA-seq (scRNA-seq) on lineage negative (Lin-) HSPC. Lin- HSPC were selected because of their functional impairment in competitive transfer experiments, and because publicly available scRNA-Seq analysis revealed that *Chtf18* mRNA levels are highest in the stem and progenitor cells in the bone marrow (**Supplementary Figures 5A, B**). *Chtf18* mRNA levels were also found to elevated in CD8 immature single positive cells (ISP), which are transitioning from the CD4-8- double negative (DN) to the CD4+8+ double positive (DP) stage of thymocyte development (**Supplementary Figure 5C, D**). The scRNA-seq analysis revealed eight clusters defined by lineage specific stem cell markers derived from the *Tabula Muris* dataset (**Supplementary Figure 6A**) (https://www.czbiohub.org/sf/tabula-muris/). The clusters showed minimal changes in representation in the *Chtf18* mut HSPC, except for the myeloerythroid progenitors (MEP), which were substantially reduced (**Figure 6A**). Pathway analysis revealed that among the differentially expressed genes (DEG), there was no enrichment for DNA-damage response pathways, but there was marked enrichment in pathways related to interferon signaling and protein folding (**Figure 6B**; **Supplementary Figures 6B, C**). Expression of the remaining clamp loader components (RFC2-5) was increased in *Chtf18*mut myeloid progenitors (**Figure 6C**). Interestingly, expression of Myc, which has been implicated in controlling HSC quiescence, was markedly elevated in *Chtf18* mut HSC/MPP (**Figure 6C**) (59). Using transcriptomic analysis to infer cell cycle status, we found that long-term HSC (LT-HSC), short term HSC (ST-HSC) and CD34 expressing LT-HSC (LT-34F) all exhibited an increase in cells in cycle (G2M/S) in the *Chtf18* mut mice, consistent with the potential loss of quiescence by HSC (**Figure 6D**).

### Analysis of the basis for impaired *Chtf18* mut HSPC function

The increased expression of *Myc* and proliferative transcriptomic signature of *Chtf18 mut* HSC raised the possibility that their failure to reconstitute hematopoiesis in the competitive setting resulted from exhaustion. To test this possibility, we performed competitive transfers using FL HSC, which are highly proliferative (60). Interestingly, *Chtf18* mut FL HSC also failed to support hematopoiesis upon competitive transfer into irradiated hosts (**Supplementary Figure 7A**). The failure of *Cht18* mut HSC to support hematopoiesis was not evident in non-competitive transfers (**Supplementary Figure 7B**), indicating that *Chtf18* mut HSPC had the capacity to support hematopoiesis but were unable to do so when forced to compete with fully competent WT HSPC. The elevated transcriptomic signatures of IFN signaling and protein folding in *Cht18* mut HSPC were reminiscent of what is observed in some mouse models of Fanconi Anemia (*Fanca-/-)* (61, 62), in which defects do not manifest until stressed (42). To test this possibility, *Chtf18* mut mice were repeatedly stressed by biweekly pIpC treatments; however, unlike the lethality and hematopoietic defects observed following serial treatment of *Fanca^-/-^* mice with pIpC, biweekly treatment of *Chtf18* mut mice had no significant impact on their viability or peripheral lymphoid populations (**Supplementary Figure 7C**). Likewise, *Cht18* mut FL HSPC grew normally *in vitro* and did not exhibit increased sensitivity to interferon-β (IFNβ), heat shock protein inhibitors (Ganetespib), or DNA damage induced by Mitomycin c (MMC) (**Supplementary Figures 7D–F**), unlike what has been observed for cells with mutations in FANC family proteins (42, 61, 62). Finally, contrary to reports in cell lines lacking CTF18, *Chtf18* mutant HSPC did not exhibit increased sensitivity to the chain-terminating chemotherapeutic agent, Cytrabine (AraC) (**Supplementary Figure 7G**) (63).

**Figure 7:**
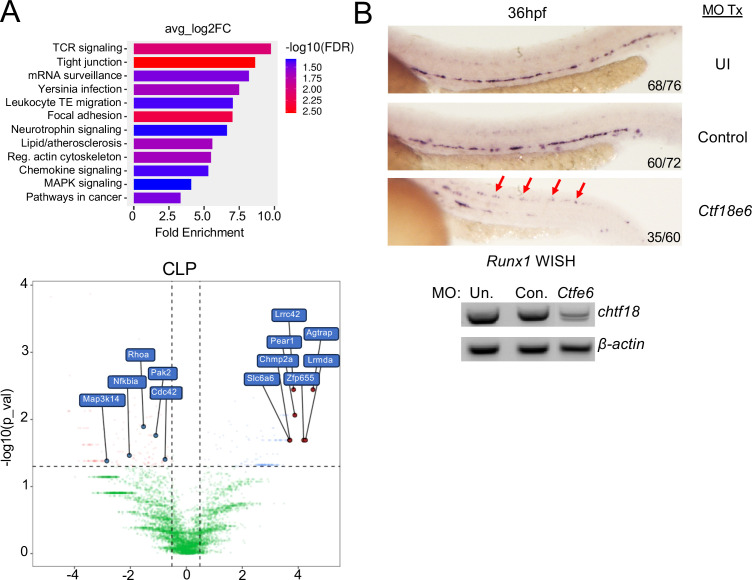
*Impact of Chtf18 variants on HSPC migration.* **(A)** Gene Set Enrichment analysis (EnrichGo) of the highly variable expressed genes between *Chtf18* WT and mut CLP cluster (upper panel). (Bottom Panel) Volcano plot of the top differentially expressed genes between *Chtf18* WT and mut CLP cluster. **(B)** The impact of Ctf18 loss on the localization of HSPC in zebrafish embryos was examined by MO knockdown of *chtf18* (*chtf18e6*), following which the location of HSPC in the caudal hematopoietic tissue was assessed by WISH using a probe for *runx1.* Statistical significance was assessed by Fisher’s Exact Test, with the p=6.8x10-7 for Con. vs Ctfe6.

Because the defect in *Chtf18* mutant HSPC did not phenocopy that seen in *Fanca-/-* mice, we re-examined the expression signature alterations in *Chtf18* mut HSPC. Interestingly, we found that *Chtf18* mut common lymphoid progenitors (CLP) exhibited changes in the expression of genes regulating cell-cell interactions, migration, and chemotaxis (**Figures 7A**; **Supplementary Figure 8A**). Specifically, the expression of several Rho family GTPases and their regulators (RhoA, Cdc42, Pak2, and Rock1) was diminished in *Chtf18* mut CLP (**Figures 7A**; **Supplementary Figure 8A**). Rho family GTPases have been shown to regulate HSC migration, and localization, in addition to playing a critical role in the asymmetric division that is required to balance self-renewal with differentiation (64–67). Indeed, loss of these effectors markedly perturbs these processes and so may explain the defective function of *Chtf18* mut HSPC in the competitive transplant setting (64–66). To determine if *Chtf18* mut HSPC caused the mis-localization of HSPC, we employed the zebrafish model, in which HSPC positioning can be readily evaluated in embryos by WISH using a *runx1* probe to identify emerging HSPC (**Figure 7B**). Indeed, we observed that knockdown of *chtf18* using MO resulted in a marked dispersal of HSPC away from the embryo midline, indicating that Ctf18 loss caused the mis-localization of HSPC (**Figure 7B**). Together, these data suggest that the competitive disadvantage displayed by *Chtf18* mut HSPC resulted from alteration of their positioning during development.

## Discussion

Here we report our investigation of the etiology of T lymphopenia in a male proband identified through newborn screening, but who was unfortunately lost to follow-up with only limited clinical and laboratory data available. Informatic analysis of WES data from the parents and proband revealed that the highest scoring variant was a component of a secondary clamp loader, *CHTF18*, a novel candidate in immunodeficiency (16). The proband exhibited compound heterozygosity of the CTF18-R751W maternal and CTF18-E851Q paternal missense variants. Functional analysis in zebrafish revealed that both mutations damaged CTF18 function and impaired their ability to support T cell development, suggesting that the proband’s T lymphopenia resulted from autosomal recessive inheritance of compound-heterozygous, damaging mutations in the *CHTF18* gene. Modeling the orthologous, compound-heterozygous mutation in mice produced a distinct phenotype. While there was no baseline deficit in T cell development specifically or more generally in hematopoiesis in the *Chtf18* mut mice, the compound heterozygous mutations in *Chtf18* abrogated HSPC function in the competitive transplant setting. The defect in function was associated with reduced expression in Rho family GTPases and their regulators, which in turn may disturb cell-cell contacts and migration of HSPC, since they exhibited altered positioning in zebrafish embryos. Taken together, these data indicate that impairing CTF18 function negatively impacts hematopoietic development through a novel mechanism that is unrelated to its canonical role as a secondary clamp loader in supporting DNA replication (16, 17).

The proband was initially identified by TREC-based newborn screening, which has both enhanced treatment efficacy for SCID and led to the identification of a number of novel immunodeficiency genes (1, 6, 9, 10, 22). While initially presenting with neonatal T lymphopenia that did not resolve in over two years, the infant experienced normal growth, and recovered from one episode of pneumonia, before being lost to follow-up. WES implicated two distinct variants with the potential to cause disease, *CHTF18 and CTBP2.* CTBP2 is a transcriptional corepressor that represses target genes, including IL-2 (48, 49), but its elimination did not impair T cell development in zebrafish. It remains possible that the two putative inactivating *CTBP2* variants in the proband could impair CTBP2 function, leading to IL-2 de-repression, which at very high levels can cause severe side effects (68); however, the patient did not manifest symptoms related to excess IL-2 in early life. In contrast, CTF18 loss in zebrafish attenuated T cell development, attesting to the importance of CTF18 function in supporting T cell development. Moreover, replacement of zebrafish Ctf18 with wild type human CTF18 restored development, indicating conservation of CTF18 function from human to zebrafish. Both the maternal (R751W) and paternal (E851Q) variants failed to complement the loss of zebrafish Ctf18 in restoring T cell development, supporting the conclusion that the patient’s compound heterozygous mutations in *CHTF18* were responsible for the proband’s T lymphopenia. Moreover, our zebrafish studies suggested that impaired T cell development might be restricted to αβ T cells after thymic seeding, which is consistent with the normal numbers and function of B and NK cells in the patient. Together, these data suggest that *CHTF18* mutations like those in this patient are more likely to be associated with T lymphopenia than SCID; however, it remains possible that other variants that more severely attenuate CTF18 function, or alter canonical functions of CTF18, might cause SCID. Finally, the block in zebrafish T cell development caused by loss of Ctf18 function was not dependent upon p53, suggesting that it was not the result of accumulated DNA damage, as might be expected if the developmental block was caused by loss of Ctf18’s support for DNA-replication as part of a clamp loader complex (53, 69).

Mice bearing orthologous mutations exhibited a different phenotype than was observed in zebrafish and our human infant, in that there was no baseline phenotype but the *Chtf18* mut HSPC were severely impaired in their capacity to reconstitute hematopoiesis in the competitive transplant setting. This may relate to the observation that MO knockdown in zebrafish can result in mosaic loss of target gene expression, creating a *de facto* competitive context similar to that seen during competitive transplantation in mice (70). Nevertheless, the murine developmental arrest was far earlier than that observed in zebrafish, in that HSPC were unable to seed the thymus or contribute to the production of any blood lineage.

We have previously found that zebrafish better modeled human disease than mice. Indeed, loss of zebrafish and human ARPC1B caused a block in T cell and thrombocyte development (15); however, Arpc1b-deficient mice do not exhibit a change in T cells or thrombocytes, but do exhibit one aspect of the anomalies seen in patients, increased IgE levels (71). Moreover, the interleukin 7 receptor alpha (IL7Rα) chain plays a critical role in T cell development in humans, mice and zebrafish. However, while IL7Rα-loss in humans and zebrafish selectively reduces T cells (72–74), IL7Rα-deficiency in mice reduces both B and T cells (75). A final example of zebrafish more faithfully modeling human disease relates to the impact of loss-of-function of PLOD2. *PLOD2* mutations in humans cause Bruck syndrome, a disease marked by bone fragility and congenital joint contractures (76), which is faithfully modeled by Plod2-deficient zebrafish that exhibit a shortened body axis and severe skeletal abnormalities with evidence of bone fragility (77). In contrast, Plod2-deficient mice are embryonically lethal due to exacerbation of ER stress and apoptosis (78). These examples emphasize how the zebrafish can more faithfully model human disease than mice bearing orthologous mutations.

Nevertheless, mouse models can serve as a useful platforms to gain mechanistic insights into the basis by which a human variant disrupts function. CTF18 is a component of a secondary clamp loader (16, 51, 53). The primary clamp loader complex comprises five components, the large RFC1 subunit and four smaller RFC subunits, RFC2-5 (16, 17). Primary clamp loaders ensure that the trimeric PCNA ring, which acts a clamp, is loaded onto DNA and functions as a landing pad for the DNA polymerase and other processivity factors required for DNA replication (16, 17). Loss of RFC complex components is lethal in yeast and higher vertebrates (18, 19). In contrast, loss of CTF18, the largest subunit of a secondary clamp loader, is not lethal in yeast or mice, though its loss does result in non-Mendelian inheritance of knockout offspring, which are smaller and have reduced testis size (20, 79). The *Chtf18* mut mice (R745W/E845Q) were born with Mendelian inheritance ratios and exhibit no defects in fertility (data not shown). Structural modeling of the *CHTF18* patient variants based on a recent cryo-EM structure of the CTF18 clamp loader suggested that the stability of the CTF18 interaction with the RFC3 subunit would be altered by R751W, thereby affecting catalysis (51); however, because E851Q was solvent facing, its impact on clamp loader function was unclear, but could affect the interaction of CTF18 and pol ∈. Nevertheless, the structural modeling will not be informative regarding the mechanism of action of the *CHTF18* variants, if the role of CTF18 is supporting hematopoiesis is unrelated to its clamp loader function.

The absence of a DNA-damage signature in the scRNA-Seq analysis of *Chtf18* mut HSPC and the normal proliferation exhibited by *Chtf18* mut FL progenitors suggests that CTF18 may be supporting hematopoiesis through activities unrelated to clamp loader function. Consistent with this idea, both CTF18 and RFC1 have also been shown to have functions independent of their clamp loading function. RFC1 can regulate NFKB activity (80) and inhibit the activity of polymerase η (Polη), while CTF18 stimulates Polη activity (69). Polη has also been shown to be enriched in actively transcribed regions of the yeast genome, so this could be a mechanism by which CTF18 could influence transcription (81). CTF18 has also been implicated in cohesin loading onto chromatin, which could influence gene expression through chromatin looping (82, 83). Finally, CTF18 has been found to associate with the nuclear pore complex, which has been implicated in gene silencing at sub-telomeric regions (84). Consequently, any of these mechanisms could be responsible for the changes in gene expression exhibited by *Chtf18* mut HSPC, including signatures for interferon signaling, protein folding, migration and RhoA GTPase signaling. The interferon and protein folding signatures were similar to those found in *Fanca-/-* mice, which also had no baseline phenotype, but exhibited HSC attrition after repeated inflammatory insults (42); however, *Chtf18* mut HSPC were not preferentially susceptible to attrition in response to those treatments. Interestingly, the expression of genes from the Rho family of GTPases, and their regulators, were reduced in *Chtf18* mut HSPC. Rho family GTPases have been shown to regulate HSC migration, localization and mobilization in addition to playing a critical role in the asymmetric division that is required to balance self-renewal with differentiation (64–67). Consequently, downregulation of these effectors is likely to explain the defective function of mouse *Chtf18* mut HSPC in the competitive transplant setting and the mis-localization of HSPC in zebrafish embryos. Specifically, CXCR4 signaling in response to CXCL12 produced by Leptin Receptor expressing cells in the perivascular niche plays a critical role in the proper positioning required to support HSC function (85–87). Thus, reduced chemokine receptor signaling may impair migration of HSC to these supportive perivascular niches, thereby depriving them of the trophic signals required for development. We have previously found that perturbation of Ccr7 and Ccr9 signaling displaced *bcl11b* mutant HSPC, although in this case their displacement resulted from increased signaling and so hematopoiesis was restored by repression of Ccr9 (9). Whether restoration of Rho family GTPase function is sufficient to reestablish the hematopoietic potential of *Chtf18* mutant HSPC remains to be tested. The impact of the *Chtf18* mut on HSPC localization caused different perturbations of hematopoiesis in mice and zebrafish, with *Chtf18* mut mouse HSPC showing no activity in the competitive setting, and the zebrafish HSPC being able to seed the thymus but not support T cell development. The basis for this difference is unclear but both defects could be the result of failure to maintain cell-cell contacts or migrate properly, given that intrathymic migration is critical for normal T cell development (88–90).

While the precise mechanism by which the *CHTF18* missense mutations could disrupt hematopoiesis and reasons for species differences in the impact of those mutations remain unclear, the analysis in zebrafish appears to align with the defect observed in the proband. Moreover, these studies highlight how the analysis of human genetic variants can uncover novel non-canonical functions for these candidate genes. Indeed, together our data identify *CHTF18* as a novel immunodeficiency gene that supports T cell development through its capacity to influence gene expression in a non-canonical manner, distinct from its traditional role as a secondary clamp loader. It remains be determined how the *CHTF18* missense mutations interfere with its noncanonical function, but this is being actively investigated.

## Data Availability

The raw data supporting the conclusions of this article will be made available by the authors, without undue reservation. WES data from the JPSCID-15 trio were submitted to dbGaP, accession number phs002968.v1.p1. scRNA-Seq data for Ctf18wt and mutant HSPC have been deposited in GEO, Accession #GSE289457; GSM8791734; GSM8791735; GSM8791736; GSM8791737.
