# Supplementary material for: Disruption of the moonlighting function of CTF18 in a patient with T-lymphopenia
Source: Front Immunol. 2025 Feb 14;16:1539848. doi: 10.3389/fimmu.2025.1539848 (PMC11868726; doi:10.3389/fimmu.2025.1539848)
Supplement: Supplementary file 1 [file DataSheet1.pdf]

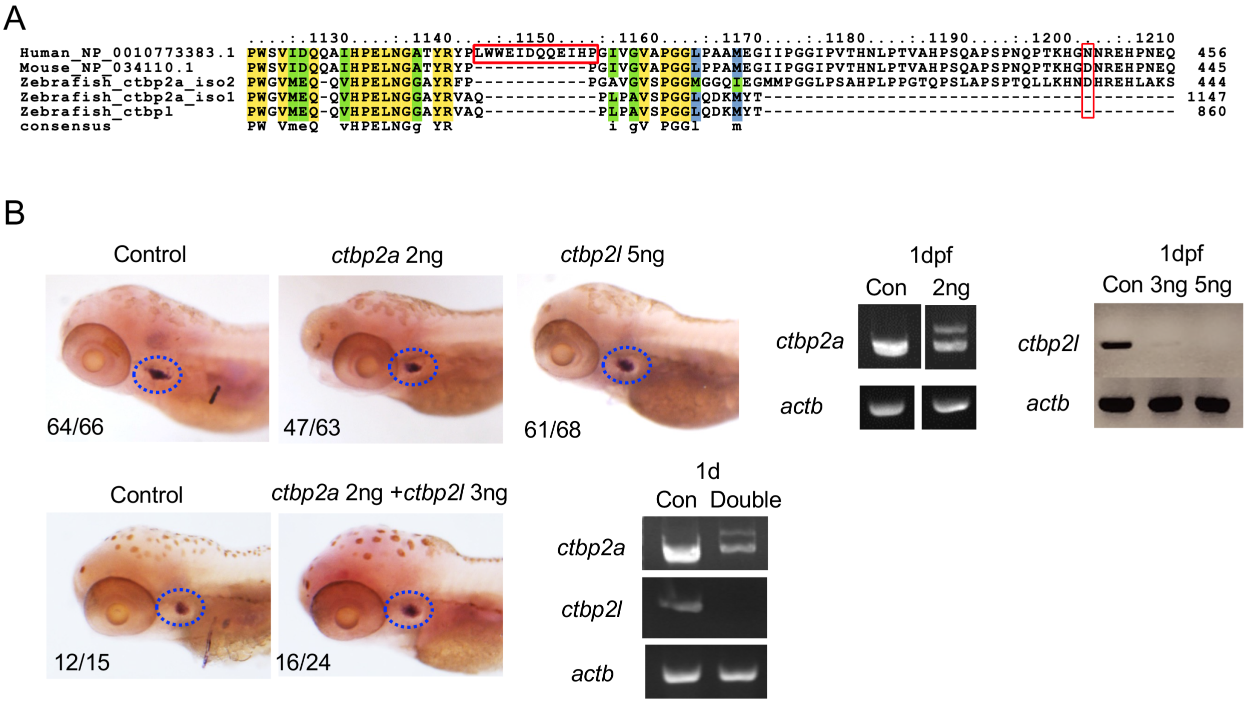


***Supplemental Figure 1. Analysis of candidate patient variant, CTBP2.*** **(A)** Sequence alignment of human, mouse and zebrafish CTBP2 paralogues. The human de novo D437N mutation and in frame 11 amino acid insertion are indicated by red square outline **(B).** *ctbp2* knockdown does not block T cell development. Zebrafish embryos were injected with MO designed to block splicing of *ctbp2a E5I6* (2ng) and *ctbp2l E1I1* (5ng) alone or in combination (2 and 3 ng, respectively). MO efficacy was verified by RT-PCR at 1dpf. The impact on T cell development was assessed by WISH using an *lck* probe at 5dpf. Thymus is indicated by dashed blue ovals.

```
Zebrafish       YFPFLPAAFHQLYAATSVPRINYPHSHYEAFTKTQHIRNALLAMLNDIPPAIQSRVCMSS
Human           YPPFLPVAFHVLFASSHTPRITFPSSQQEAQNRMSQMRNLIQTLVSGIAPATRSRATPQA
Mouse           YLPFVPAAFHVLFASSHVPRITFPSSQQEAQTRMSQTRNHIQTLVSGMAPTTRSRATPQA
                * **:*.*** *:*:: .***.:* *: ** .: .: ** : :::..: *: :**.  .:

Zebrafish       LCLDILSLLLELISPKLRPVNPQLYSTREKQQLYDLIDTMINYNLTYRQDRTPEGQYTYV
Human           LLLDALCLLLDILAPKLRPVSTQLYSTREKQQLASLVGTMLAYSLTYRQERTPDGQYIYR
Mouse           LVLDTLCLLLDVLAPKLRPVSTQLYSAHEKQQLSCLVGTMLAYSLTYHQERTPDGQYLYK
                * ** *.***::::******. ****::*****  *:.**: *.***:*:***:*** *

Zebrafish       LEPNIEDVVNYPGLPPRRQLTYQVKQLIARETELERMRRVERAQQSRNPQKMETVNKNQ-
Human           LEPNVEELCRFPELPARKPLTYQTKQLIAREIEVEKMRRAEASARVENSPQVDGSPPGLE
Mouse           LEPNVEEVCRFPELPARKPLTYQAKQLIAREIEMEKMRRAEALAWARSGPQVDQGSSGPA
                ****:*:: .:* ** *: ****.******* *:*:***.*     ..  :::    .

Zebrafish       ----EEKKTEVKKQTSRNHQQRLENIVKQTVVETRPELDFFGRAIVPKEKPVVTATSEDG
Human           GLLGGIGEKGVHRPAPRNHEQRLEHIMRRAAREEQPEKDFFGRVVVRSTAVPSAGDTAPE
Mouse           SLWTDSGEKGTRQPVPRNHEQRLEHIMTRATVQEQPERDFFGRVVIRKVAVPSREVEAPQ
                       :. .:: . ***:****:*: ::. : :** *****.:: .

Zebrafish       KVSGVLNIGKAVGNSDVWFRFNEGVSNAVRRNVYIRELL
Human           QDSVERRMGTAVGRSEVWFRFNEGVSNAVRRSLYIRDLL
Mouse           KDADEWRMGVAVGRSEVWFRFNEGVSNAVRRSLYIRDLL
                : :   .:* ***.*:***************.:***:**
```

***Supplemental Figure 2. Sequence alignment of CHTF18 orthologs.*** Full length human, mouse and zebrafish *CHTF18* were aligned using ClustalX. Conservation across the sequences is indicated (identical-*, highly similar-:, similar-.) with the conserved patient variant amino acids boxed in red.

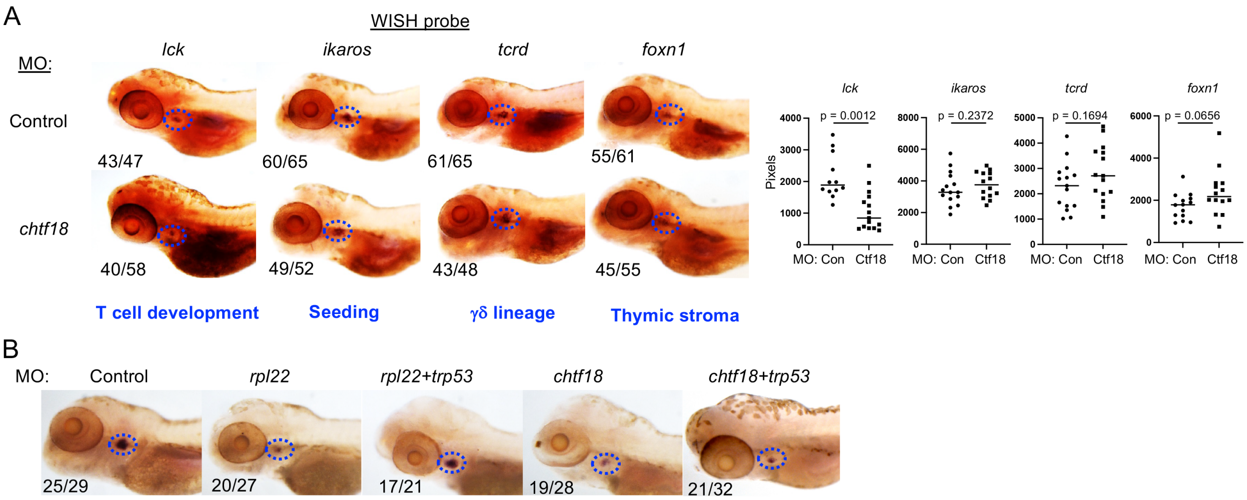


***Supplemental Figure 3. Analysis of distinct thymic cell subsets in chtf18 morphants.*** **(A)** The effect of *chtf18* knockdown on development of: αβ T cells, *lck;* seeding progenitors, *Ikaros;* γ δ T cells, *tcrd;* and thymic stroma, *foxn1.* WISH signals were quantified using Image J and depicted graphically. Statistical significance was evaluated using two way ANOVA. p values are indicated on graphs. **(B)** The dependence of the developmental arrest in *chtf18* morphants on p53 was assessed by knocking down *Trp53* using splice site MO. *Rpl22* served as a positive control. The impact of T cell development was assessed by lck WISH and the thymus is marked by dashed blue ovals. The frequencies of embryos with the depicted staining pattern are indicated on the images.

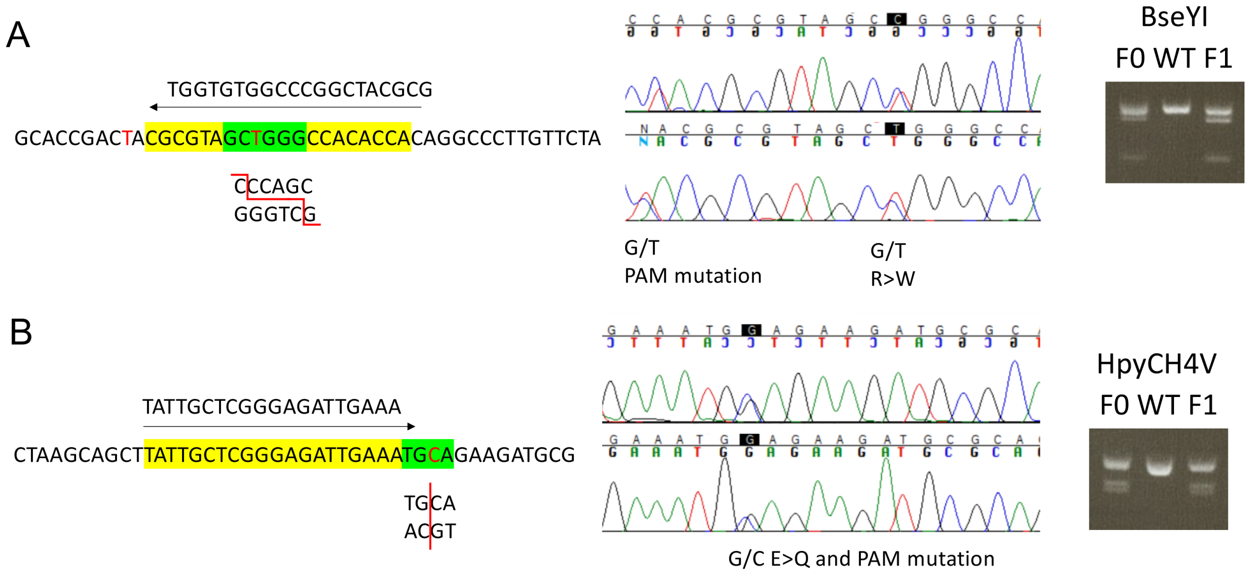


***Supplemental Figure 4. Generation and sequence validation of Chtf18 mutant mice.* (A, B)**. Mice bearing mutations equivalent to the patient variants *Chtf18*R745W **(A)** and *Chtf18E845Q* **(B)** were produced by CRISPR mediated gene editing. sgRNA target sequences are shown with the sgRNA sequence indicated, its target region highlighted in yellow, mutations in red, and newly created restriction enzyme sites indicated in green. Sequence trace files showing the protospacer adjacent motif (PAM) mutation and specific coding mutation are depicted, as are the restriction digests employed for typing founder (F0) and progeny (F1) tail DNA.

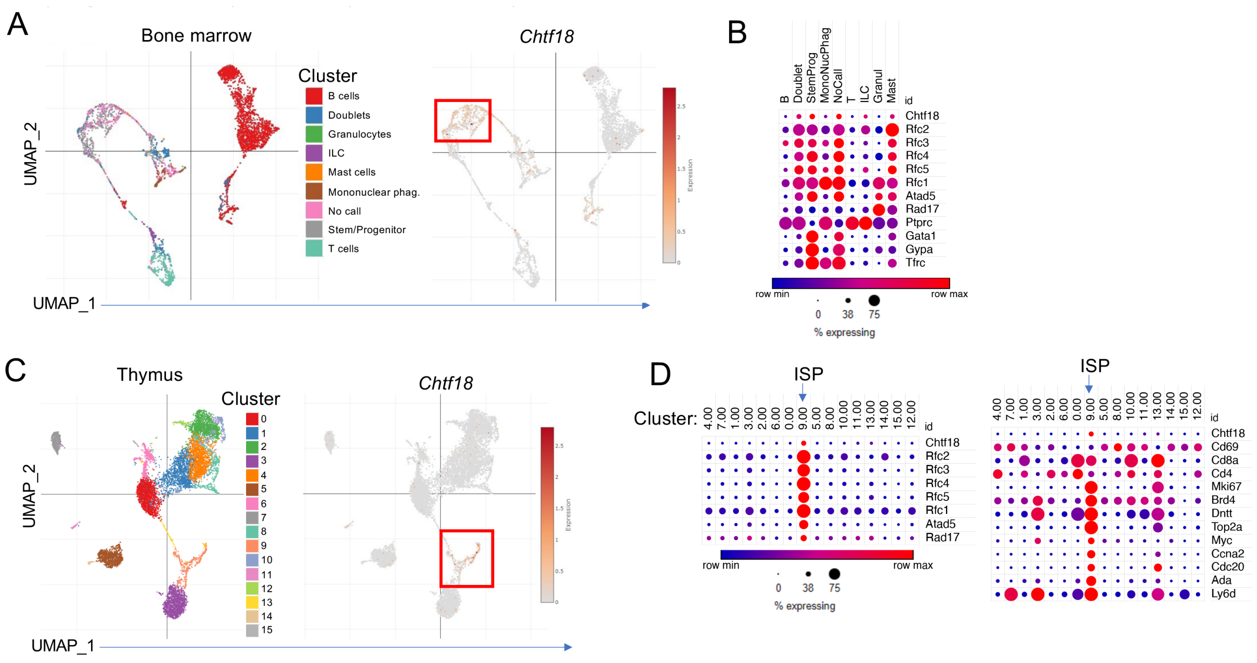


**Supplemental Figure 5. *Chtf18 expression in thymus and bone marrow progenitors by public scRNA-Seq data*.** **(A)** (left) UMAP plot of 9 clusters resulting from publicly available scRNA-Seq analysis of adult C57BL/6 mouse bone marrow (3865 cells). (right) *Chtf18* expression mapped onto the UMAP coordinates within the clusters with white to deep red indicating expression. **(B)** Bubble plot of the scaled expression of clamp loader and lineage specific factors in each cluster with minimum expression shown in blue and maximum in red. Bubble size indicates the percentage of cells in each cluster expressing the factor. **(C)** (left) UMAP plot of 16 clusters resulting from publicly available scRNA-Seq analysis of adult C57BL/6 mouse thymus (10,741 cells). (right) *Chtf18* expression mapped onto the UMAP coordinates within the clusters with white to deep red indicating expression. **(D)** Bubble plot of the scaled expression of clamp loader and lineage specific factors in each cluster with minimum expression shown in blue and maximum in red. Bubble size indicates the percentage of cells in each cluster expressing the factor.

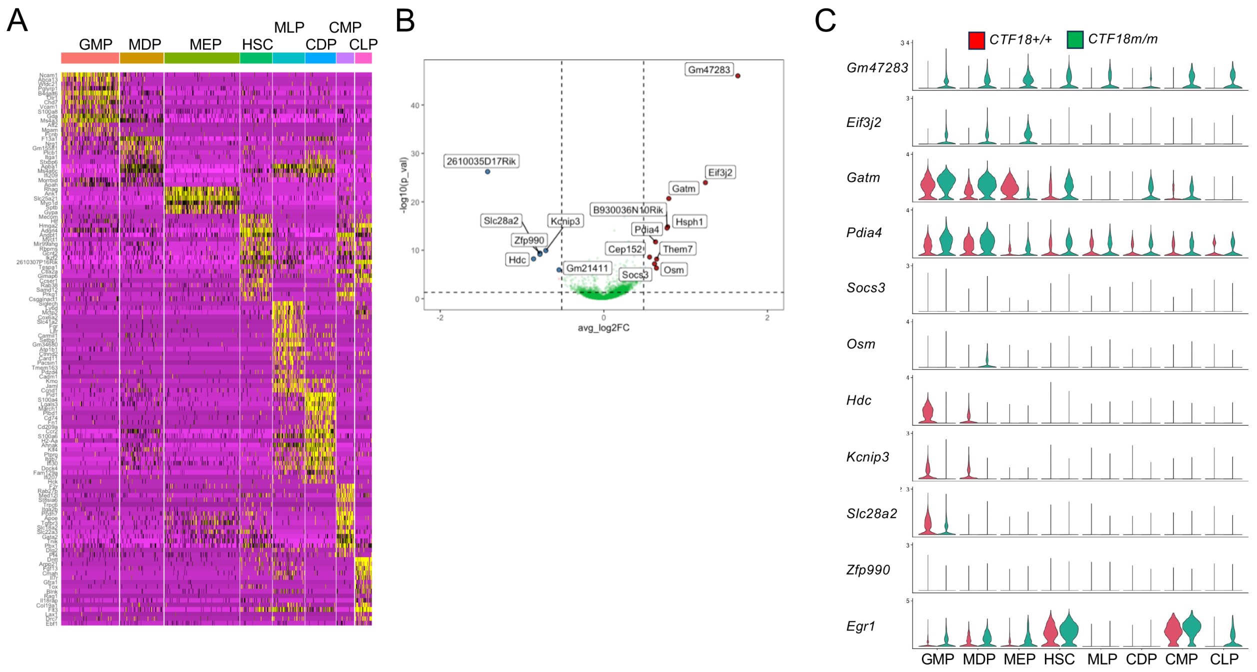


**Supplemental Figure 6.** ***scRNA-Seq analysis of Chtf18 mut HSPC.***

A) Heatmap of the top differentially expressed genes in each of the eight HSPC clusters in *Chtf18* mut mice. B) Volcano plot of the top differentially expressed genes between *Chtf18* wildtype and mut HSC/MPP cluster. C) Violin plot representation showing gene expression levels of the top upregulated genes across all eight clusters in *Chtf18* WT and mut HSPCs.

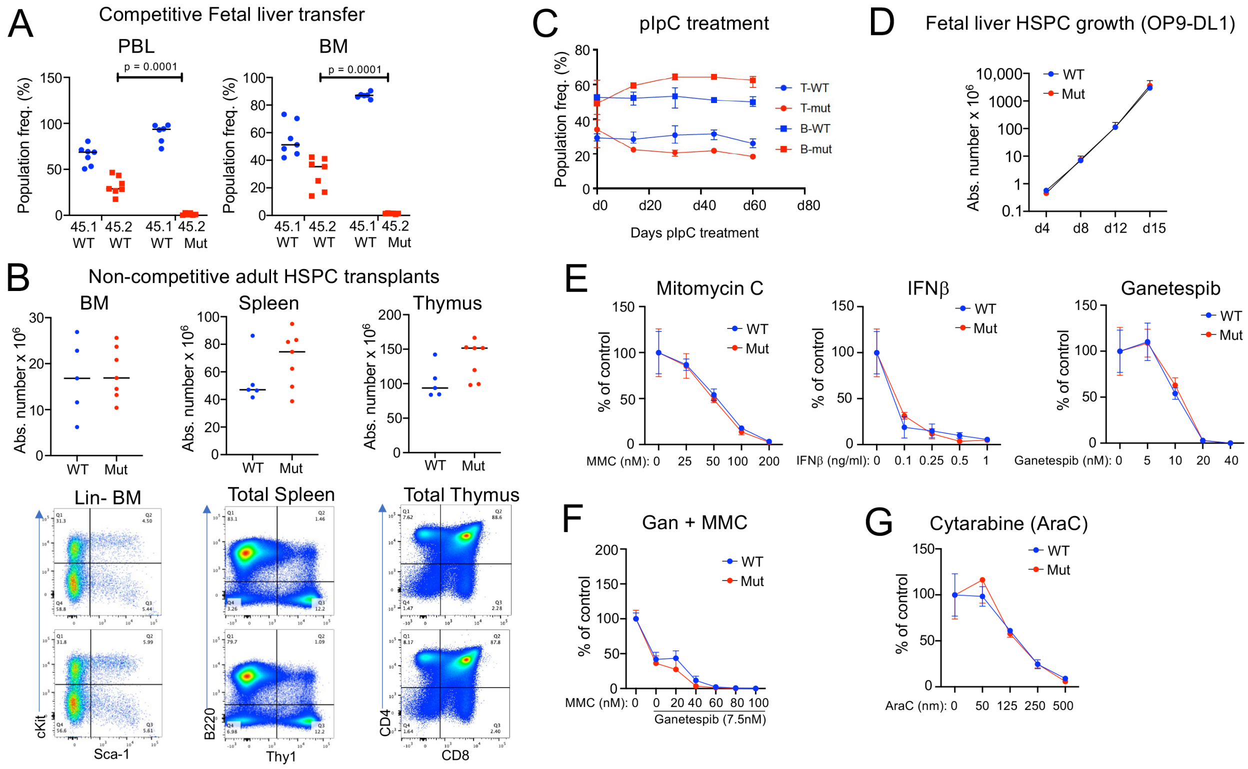


**Supplemental Figure 7.** ***Function of adult and fetal Chtf18 mut HSPC.*** **(A).** The ability of day 14.5 FL HSPC from WT and *Chtf18* mut mice (CD45.2) to repopulate hematopoiesis was assessed by competitive transplantation using allotype marked (CD45.1) competitor. 100,000 HSPC from CD45.2 WT or *Chtf18* mut mice were mixed with an equal quantity of CD45.1 competitor cells and transferred into irradiated recipients. After 6 weeks, the hematopoietic reconstitution was assessed on peripheral blood lymphocytes (PBL) and on explanted bone marrow cell suspensions by flow cytometry using anti-CD45.1 and anti-CD45.2 antibodies. Cell frequencies were displayed graphically as scatter plots. Statistical significance was assessed by two-way ANOVA. **(B).** The ability of 100,000 adult WT and *Chtf18* mut HSPC (CD45.2) to repopulate hematopoiesis upon transfer to irradiated (CD45.1) recipients in a non-competitive setting was assessed on the indicated explanted tissues by flow cytometry after 6 weeks. Cell frequencies were displayed graphically as scatter plots. Statistical significance was assessed by two-way ANOVA. **(C).** The capacity of WT and *Chtf18* mutant HSPC to withstand repeated cycles of inflammation-induced activation was assessed by serial treatment with pIpC. The mice were dosed with 15mg/kg pIpC two times per week for 60 days and the impact on lymphopoiesis was assessed by flow cytometry on PBL at the indicated time points. Statistical significance was

assessed by two-way ANOVA. **(D-G)** The growth of WT and *Chtf18* mut FL precursors on OP9-DL1 monolayers **(D)** and their ability to withstand treatment with **(E-F)** interferon-β, DNA-damaging agents (Mitomycin C), a HSP90 inhibitor (Ganetespib), and (**G)** Cytarabine was assessed on triplicate cultures after 4d by cell counting. Results of dose-response analysis were depicted graphically. Statistical significance was assessed by multiple unpaired t tests.

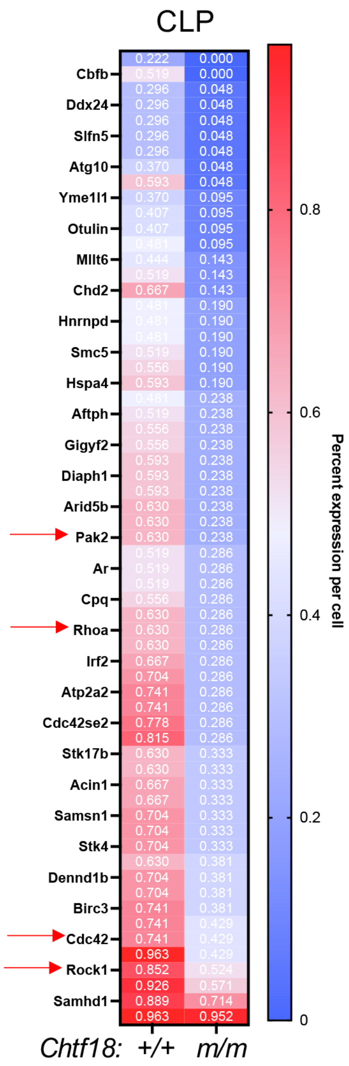


**Supplemental Figure 8.** ***Impact of Chtf18 mutations on gene expression in CLP.*** A heat map of genes differentially expressed in CLP is depicted. Rho family GTPases and their regulators are denoted by red arrows.
